# An outlook into the flat land of 2D materials beyond graphene: synthesis, properties and device applications

**DOI:** 10.1088/2053-1583/abc13d

**Published:** 2020

**Authors:** Amber McCreary, Olga Kazakova, Deep Jariwala, Zakaria Y Al Balushi

**Affiliations:** 1 Nanoscale Device Characterization Division, Physical Measurement Laboratory, National Institute of Standards and Technology, Gaithersburg, MD 20899, United States of America; 2 Department of Quantum Technology, National Physical Laboratory, Hampton Road, Teddington TW11 0LW, U.K; 3 Department of Electrical and Systems Engineering, University of Pennsylvania, Philadelphia, PA 19104, United States of America; 4 Department of Materials Science and Engineering, University of California, Berkeley, CA 94720, United States of America

**Keywords:** synthesis, characterization, devices, properties, magnetism

## Abstract

The field of two-dimensional (2D) and layered materials continues to excite many researchers around the world who are eager to advance and innovate viable routes for large scale synthesis, doping and integration of monolayers and the development of unique characterization approaches for studying and harnessing exotic properties that will enable novel device applications. There has been a large interest in 2D materials beyond graphene, with particular emphasis on monoelemental materials (phosphorene, silicene, tellurene, *etc.*), 2D compounds (MXenes, oxides, nitrides, carbides and chalcogenides), their alloys and layered van der Waals heterostructures. This is not only indicated by the significant increase in the number of peer reviewed publications each year in this area of research, but also by the surging number of conference sessions focusing on 2D materials beyond graphene. This Perspective article highlights some of the recent advances in the field from a diverse international community of theoretical and experimental researchers who participated in the symposium ‘Beyond Graphene 2D Materials—Synthesis, Properties and Device Applications’ at the Materials Research Society (MRS) Fall 2019 meeting.

## 1. Introduction

The isolation of graphene from bulk graphite into devices in 2004 ignited the field of two-dimensional (2D) and layered materials, which has since rapidly expanded in the avenues of synthesis, discovered properties and novel applications [1]. Although graphene has been one of the first manufacturable 2D materials to be realized on an industrial scale, it has been only the tip of the iceberg in the flat land of (layered) 2D materials. A plethora of materials, both experimentally discovered and theoretically predicted, have weak van der Waals (vdW) interactions governing their interlayer coupling. Thus, these materials can be produced and maintain freestanding nature at thicknesses of individual unit cells using both top-down and bottom-up approaches. The multitude of 2D materials plus variability in regard to composition, crystal structure, and layer thickness leads to a variety of resulting material properties, including semiconducting, metallic, insulating, superconducting, magnetic, and topological, covering all of the components necessary to create electronic, opto-electronic, and spintronic devices [2–6]. The weak interlayer coupling also allows the stacking and twisting of multiple 2D layers into vertical heterostructures without lattice mismatching being a limiting factor in the quality of the structure.

In this Perspective, we provide a view into the latest developments in this area of research focusing on results presented in Symposium FF01 ‘Beyond Graphene 2D Materials—Synthesis, Properties and Device Applications’ at the 2019 Materials Research Society (MRS) Fall Meeting, held in Boston, USA and featuring over 110 talks and 175 poster presentations on recent work in synthesis, properties, and device applications of these materials. As one of the most impactful symposia on 2D materials at MRS, it brought together a diverse scientific community of interdisciplinary researchers from materials science, physics, chemistry, and electrical engineering. The first section of this Perspective discusses the synthesis of 2D materials, including fundamental limits on quality, scaling solutions to push 2D materials beyond the flake limit, novel approaches to synthesis, and the rise of 2D magnetism. The second section highlights advances in characterization; from optical microscopies and spectroscopic methods to scanning probe techniques and methods for probing the properties of 2D magnets. In the third section, we focus on recent developments on novel devices, such as logic and memory, photonic and opto-electronic devices, and sensors enabled by 2D materials. Finally, a future outlook is provided.

## 2. Recent outlook on synthetic 2D materials

The design space for new synthetic 2D materials beyond graphene continues to expand, allowing researchers to explore uncharted opportunities in creating novel heterostructures. Here, we highlight some of the recent developments in the field of 2D synthesis and discuss remaining challenges, with an outlook into what is to come:

### 2.1. High quality 2D crystals

**Figure 1.**
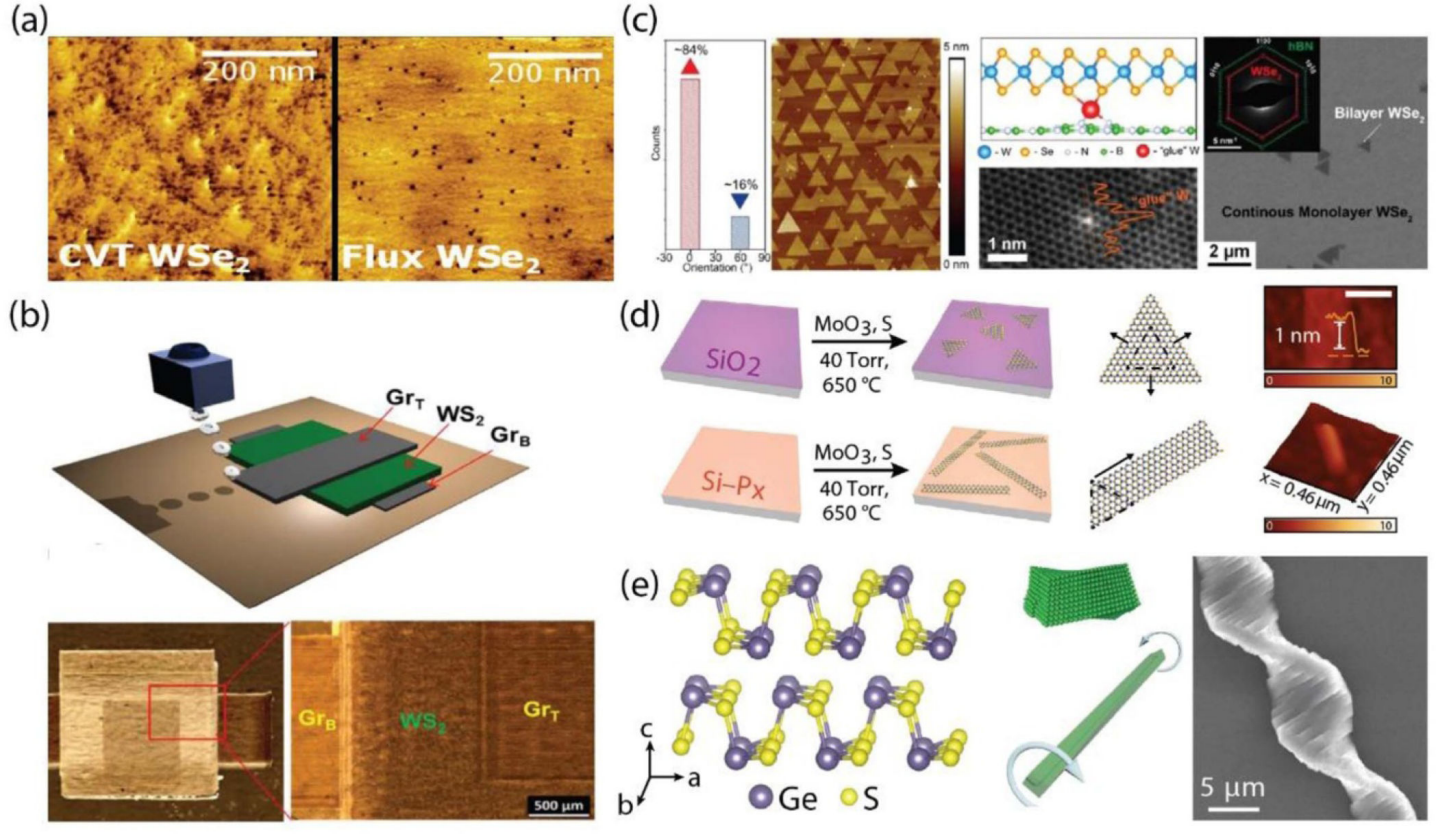
(a) STM topography image comparing the defect density of CVT and flux-grown-WSe_2_ bulk crystals (adapted with permission from reference [7]). (b) Schematic and optical image of an all-printed heterostructure photodetector on a paper substrate, showing the graphene top and bottom electrodes with WS_2_ sandwiched between them (adapted from reference [21]). (c) Histogram of AFM highlighting the defect-controlled nucleation and orientation of WSe_2_ on hBN. The schematic, TEM, SEM micrographs and selected area electron diffraction (SAED) illustrates how breaking the degenerate 0° and 60° orientation leads to single orientated domains (adapted with permission from reference [29]). (d) Schematic of the substrate-directed synthesis of 1D MoS_2_ *via* a PH_3_ surface pre-treatment and corresponding AFM images (adapted with permission from reference [30]). (e) Schematic of GeS crystal twisting morphology as seen in the SEM on the right (adapted from reference [36]).

Recent advances in synthesis have led to the growth of bulk crystals of 2D materials with intrinsic physical properties approaching their fundamental limits, particularly in regard to reducing point defects in transition metal dichalcogenide (TMD) 2D semiconductors. For example, TMD crystals produced by the self-flux technique showed dramatically lower defect densities compared to crystals grown by the chemical vapor transport (CVT) method [7], as shown in figure 1(a). These recent efforts provide plausible pathways towards improving the basal plane quality for devices. Since the pioneering works carried out at the National Institute for Materials Science (NIMS), Japan, on the synthesis of high-quality hexagonal boron nitride (hBN) crystals, encapsulation of 2D materials with exfoliated hBN single crystal flakes has been the standard in the research community, leading to many high-impact studies and a variety of device applications to date [8]. Recently, there has been an interest in isotopically enriched crystals of hBN produced from molten metal solutions. Unlike the crystals produced by the NIMS group, these crystals are grown at atmospheric pressures, providing an effective route to produce large, monoisotopic, high purity ^10^B and ^11^B enriched hBN single crystals with enhanced polariton lifetimes for applications already demonstrated in nanophotonics and optics, to name a few [9–11]. New insights have been recently revealed in this bulk crystal growth process, particularly a better understanding of the solubility of nitrogen in the boron-containing melt with the addition of additives [12–14]. This understanding provides routes to optimize the atmospheric crystal growth process for higher quality hBN crystals with a final crystal size and defect density comparable to the NIMS-grown crystals available today.

### 2.2. Solution processing as a viable route to scaling

There are a wide variety of strategies for exfoliation of 2D materials demonstrated to date. Beyond mechanical exfoliation of bulk crystals, liquid phase exfoliation presents a viable solution for mass production of monolayer flakes that can be processed into films using standard low-cost, scalable fabrication technologies such as spin coating and roll-to-roll manufacturing [15]. To control the thickness and lateral size of the exfoliated sheets, practical considerations on the role of surfactants and dispersing solvent medium as well as control of concentration and viscosity of dispersed exfoliated 2D materials are needed [16–18]. Another important consideration in liquid phase exfoliated bulk crystals is the high concentration of basal plane and edge defects that act as recombination sites, thus limiting the materials’ performance for device applications. Strategies to mitigate defects in liquid phase exfoliated TMDs have been designed through surface passivation techniques, achieving internal quantum efficiency for photon harvesting similar to that of mechanically exfoliated flakes [19]. It is also possible to transform dispersions of 2D materials into inks suitable for printable electronics (figure 1(b)). Recently, 3D printed electrodes using highly concentrated, water-based inks were demonstrated from liquid phase exfoliated flakes for micro super-capacitors and a variety of devices including photosensors and logic devices [19, 20]. In addition, developing 3D architectures using 2D material inks as building blocks is now possible. This provides a new degree of material complexity with direct ink writing strategies that contribute to the already booming field of additive manufacturing. More importantly, the mechanical flexibility and biocompatibility of 2D materials has gained significant traction in all-printed-based heterostructures for wearable tattoo devices. To print such device structures, control over mixing between printed 2D crystals is critical, as uncontrolled interfaces can perturb device performance [21–23].

### 2.3. Scaling 2D single crystals beyond the flake

Beyond bulk crystal growth of hBN, discussions surrounding the synthesis of hBN by chemical vapor deposition (CVD) are of prominent interest in the 2D community, as hBN layers are vital substrates for growth of other ‘electronic-grade’ 2D semiconductors [24]. In particular, there has been a large emphasis on the use of carbon-free sources, such as diborane, to produce wafer scale, single crystal monolayers of hBN [25]. More recently, metalorganic chemical vapor deposition (MOCVD) has emerged as a promising method for the synthesis of wafer-scale films of monolayer 2D materials beyond graphene, such as TMDs, which was first reported back in 2015 on SiO_2_ [26]. However, the misorientation and reduced domain size of TMDs grown on SiO_2_ substrates as well as the long growth times remain limitations of this process. MOCVD is an empirical science guided by thermodynamics and kinetics, both of which are critical to understand in order to achieve the necessary control over nucleation and the domain shape and size. Insights on the science and practice of MOCVD in regard to ‘epitaxial’ growth of TMD films is nicely summarized in a review article, where practical considerations including reactor design, precursor selection, growth conditions and choice of substrate are discussed [27]. Researchers in the community have also been placing a large emphasis on utilizing carbon-free precursors for TMD growth, specifically the use of H_2_S and H_2_Se liquefied toxic gases [28]. In addition, the influence of conditions to achieve lateral *versus* vertical growth are important considerations to get to high quality, single crystal monolayer films. Recently, a defect-mediated nucleation and orientation-controlled growth process of WSe_2_ on hBN, shown in figure 1(c), resulted in domains of WSe_2_ with a preferred single orientation of over 95% [29]. This led to a reduction in the density of inversion domain boundaries (IDBs), which result from the coalescence of random, degenerate 0^*°*^ and 60^*°*^ domains that are commonly observed during the growth of TMDs on sapphire. The misorientation of the domains also reduces the degree of spin and valley polarization in large area grown films. Thus, breaking this degeneracy has implications in producing homogenous monolayer films with distinct spin and valley-dependent optical selection rules that have mainly been observed in mechanically exfoliated TMD flakes and individual domains of grown synthetic materials. Moreover, a novel approach to control the orientation and dimensionality of TMDs has recently been reported by pretreating the surface of Si (001) with phosphine (PH_3_) [30]. The phosphorus-reconstructed Si surface directly influences the dimensionality of sequentially CVD-grown MoS_2_ (figure 1(d)). Nanoribbon structures were produced with systematic control over their widths (between 70 nm and 500 nm) by changing the concentration of PH_3_ introduced during the pre-treatment step.

As an alternative to CVD, molecular beam epitaxy (MBE) has been demonstrated as a promising growth technique. The use of MBE enables the growth of high-quality, 2D layered transition metal ditelluride and diselenide monolayers through the combination of high purity elemental sources and the ultra-high vacuum (UHV) growth environment [31]. Advanced *in situ* material characterization techniques, such as reflection high-energy electron diffraction and low-energy electron diffraction, in UHV have been key enablers to the fundamental understanding of the growth thermodynamics and kinetics of monolayer films by MBE to ultimately achieve atomically sharp vertical vdW heterostructures. Unlike with MBE, the control over layer number and order of any combination of 2D materials with CVD has not fully come to fruition, at least not for wafer-scale areas. Control over this is critical, as the layer number plays a significant role in the overall physical properties of a grown vdW heterostructure. However, the growth of high quality, sulfur-containing TMD monolayers still remains elusive with MBE.

### 2.4. New approaches to 2D synthesis

High-throughput computational materials screening remains an active area of research to efficiently explore the composition space for 2D materials [32]. While the list of predicted 2D structures continues to grow rapidly, experimental synthesis of these newly identified structures is lagging behind. This is partially due to our inability to kinetically obtain the predicted phases using conventional synthesis routes, namely CVD, MOCVD, MBE and solution-based approaches. Regardless of these constraints and the limited number of 2D material systems studied thus far, interest in computationally predicted materials continues to expand. One new approach to synthesis takes advantage of the interface of epitaxial graphene on SiC. This interface provides a unique materials design space for 2D compounds and metals confined at heterointerfaces [33, 34]. The ability to reduce the dimensionality to their atomic limit, such as in the case of 2D metals, has the potential to enable new or enhance existing phenomena for applications in quantum science and sensing. Moreover, the stabilization of other 2D materials at a heterointerface has been vital in the formation of unique materials such as monolayer tellurene, a 2D phase of tellurium [35]. This has been achieved through a wafer-bonding process between the interface of two CdTe substrates. The single atomic layer of tellurene stabilized between the CdTe heterointerface has been revealed to be metallic, containing Dirac-cone-like features with significant asymmetric spin-orbit splitting.

Other novel vdW structures recently reported include the successful synthesis of 1D vdW MoS_2_ and GeS using the vapor-liquid-solid mechanism as well as the synthesis of 1D helical vdW crystals with discretized Eshelby twist [36–39]. These examples represent the forefront of a unique opportunity to manipulate the topology in vdW structures and provide a new degree of freedom to tailor their physical properties (figure 1(e)). Furthermore, a variety of emerging hybrid organic-inorganic 2D heterostructures such as 2D layered halide perovskites and superlattices from monolayer 2D polymers have been successfully synthesized [40, 41]. These 2D systems take advantage of the tunable functionalities of molecular building blocks by structuring them into atomically thin layered materials. Finally, layered ceramic metal carbides and carbonitrides, known as MXenes phases, have garnered significant interest since their discovery in 2011 [42]. MXenes are produced through the removal of the (A) unit from the bulk MAX phase, typically *via* selective etchants, concentrated hydrofluoric acid (HF) being among the most conventionally utilized. The formula of MXenes are denoted as ${\text{M}}_{\text{n}+1}{\text{X}}_{\text{n}}{\text{T}}_{\text{x}}$, where M is an early transition metal, X is carbon and/or nitrogen, T represents the surface termination and n is the number of terminating groups per formula unit [43]. Among the classes of various MXene families are Ti_3_C_2_T_*x*_ phases which inherit superior conductivity (4600 ± 1100 S cm^*−*1^), high volumetric capacitance (∼1000 F cm^*−*3^) and good environmental stability [44, 45]. Careful surface termination with O,–OH,–Cl and/or –F groups has opened up the opportunity for large-scale production of a diversity of other stable MXenes phases for a variety of applications, most prominently in areas of energy such as electrodes, supercapacitors and electromagnetic interference (EMI) shielding [43, 46–49].

### 2.5. The rise of 2D magnetism

Ever since the discovery of long-range magnetic ordering in atomically thin, vdW materials in 2017, research into 2D magnetism has rapidly expanded [50, 51]. Different vdW magnets display a wide variety of spin ordering and phenomena, including ferromagnetic (FM) semiconductors/insulators (*i.e.* CrI_3_, Cr_2_Si_2_Te_6_, Cr_2_Ge_2_Te_6_), FM metals (*i.e.* MnSe_2_, VSe_2_), itinerant FMs (Fe_3_GeTe_2_), and insulating antiferromagnets (AFM) such as transition metal phosphorus trichalcogenides (*e.g.* FePS_3_, MnPS_3_, NiPS_3_, MnPSe_3_) [52, 53]. Even within just the chromium trihalide family Cr*X*_3_ (*X* = I, Br, and Cl), the spin direction, interlayer magnetic ordering, exchange gap, magnetic anisotropy, and magnon excitations intricately depend on the halogen atom [54]. Magnetic ordering temperatures in these 2D magnets range from those requiring liquid helium (*e.g.*, Cr*X*_3_, Cr_2_*X*_2_Te_6_) to those achievable with liquid nitrogen (*e.g.* FePS_3_, NiPS_3_), and some of the FM metals even have long-range ordering at room-temperature (MnSe_2_, VSe_2_) [53–55]. CrI_3_ is one of the most well studied 2D magnets due to the AFM interlayer stacking in atomically thin samples that results in layer-dependent magnetism, with non-zero (zero) magnetization present in samples with odd (even) number of layers [50]. The additional tunability of the magnetic state *via* applied electric and magnetic fields introduces new opportunities in spintronics and other magneto-optoelectronic devices, including spin tunnel field-effect transistors [56, 57]. While there are a few reports on the growth of 2D magnets using CVD and MBE [58–65], at present most of the research into 2D magnetism relies on mechanical exfoliation of bulk crystal grown *via* CVT methods. The synthesis of 2D magnetic crystals is in its infancy and will need to face an uphill battle that includes enabling air stability and ensuring synthesis of the appropriate crystal phase.

Although the intrinsic 2D FM semiconductors listed above show great promise in proof-of-concept devices for spintronics, their Curie temperatures are below room temperature and some of the materials are plagued by dramatic air sensitivity, both of which can significantly limit their potential applications. Thus, a key research goal is to realize an air-stable, atomically thin material with long-range FM above room temperature that simultaneously possesses semiconducting nature with gate tunability. In this sense, dilute magnetic TMD semiconductors that can create spin-polarized currents are being investigated, yet the difficulty lies in preventing interstitial substitutions, clusters, or alloy formations during the growth. Small amounts (0.1%–1%) of vanadium doping in WSe_2_ monolayers grown by CVD has been recently shown to display room temperature FM domains, while also retaining the p-type semiconducting nature of WSe_2_, with high ON/OFF current ratios on the order of 10^5^ [66]. No vanadium aggregation was observed in high resolution scanning transmission electron microscopy, and because the FM order is established through free hole carriers, the magnetic ordering can be clearly tuned *via* a back-gate bias [66–68]. Other examples of dilute magnetic semiconductors based on TMDs recently reported include Mn in MoS_2_, Fe in MoS_2_, and V in WS_2_ [69–71]. Current growth techniques are limited to single crystal, monolayer islands approximately tens of microns in size, where future work towards scalable growth and larger magnetic domain sizes is necessary. Nevertheless, this work is a step forward to investigate practical applications of TMDs in spintronic devices operating near room temperature.

## 3. Advances in characterization of 2D materials

The recent progress in the synthesis of 2D materials and their heterostructures have generated the need to develop new, non-destructive characterization methods to fully explore the fascinating properties of these atomically thin materials. Ideal characterization techniques of 2D materials should be non-invasive with single-atomic-layer thickness and (sub-)nanometer lateral resolution. From a commercial perspective, however, fast and large area scanning capabilities are desired. While the quality of large area grown 2D materials has improved over the years, these materials still host different types of defects such as vacancies, grain boundaries, edges, and impurities, all of which strongly influence the physical properties of the materials. The development of robust, multidimensional, high-throughput, and large-scale characterization techniques sensitive to such defects is crucial for the establishment of integrated technologies and material growth optimization. There has been a rapid development in characterization techniques that have been extensively covered in many articles and reviews, with strong emphasis on scanning probe microscopies to correlate morphology and structural quality of monolayer and few-layer 2D films [72], for example when combined with in-plane x-ray diffraction characterization [73]. Here, we highlight recent examples applied to beyond graphene 2D materials from this MRS symposium.

### 3.1. Optical microscopies

**Figure 2.**
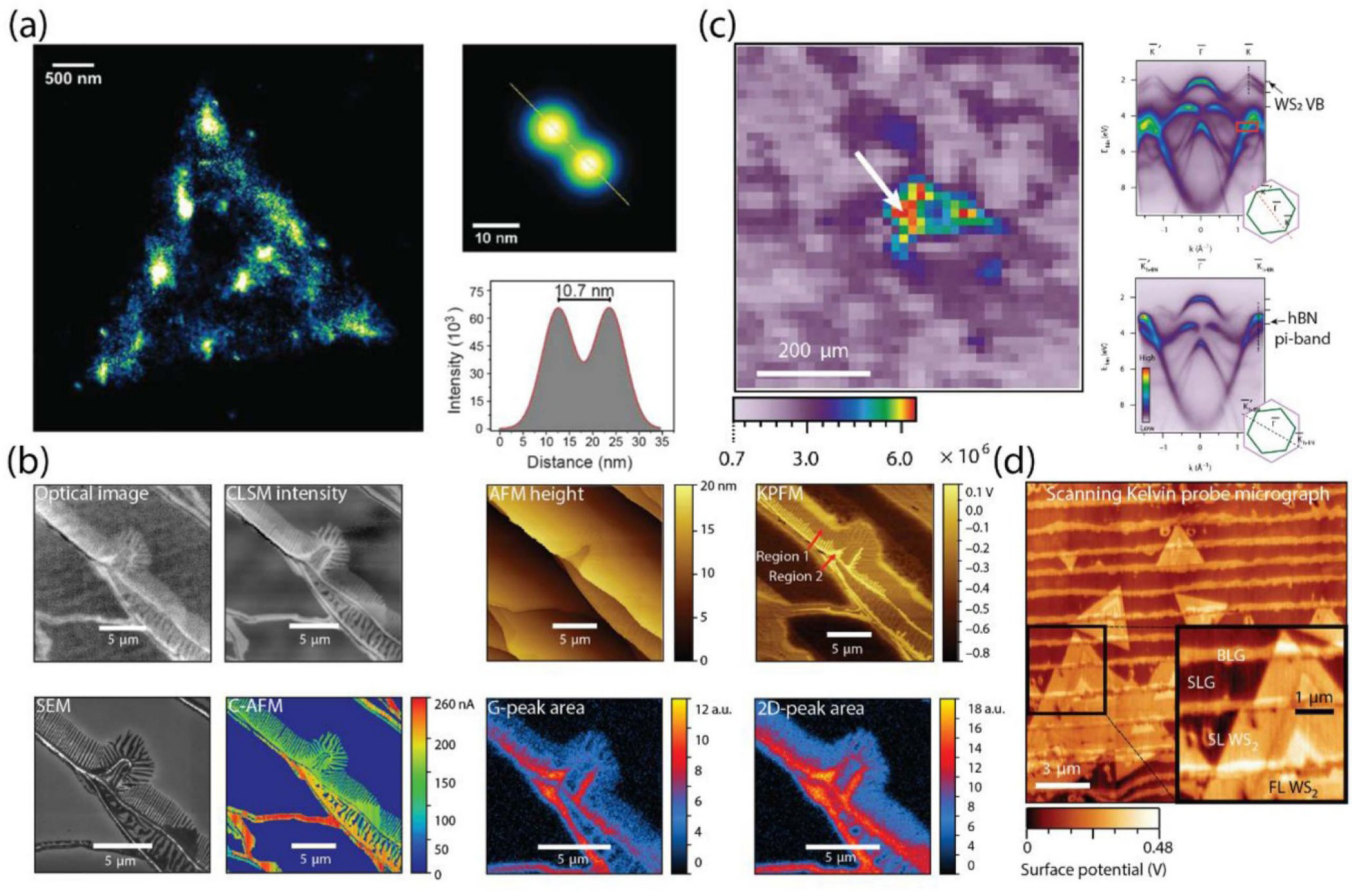
(a) Left: super-resolution image of optically active defects in hBN; right: two best resolved emitters with a distance of 10.7 nm (adapted with permission from reference [76]). (b) Graphene nanoribbons on SiC characterized by various methods as indicated in the figure (adapted with permission from reference [78]). (c) Spatially resolved electronic structure mapping of a WS_2_/hBN heterostructure. Left: Spatial map of photoemission intensity; right: ARPES of the single-layer WS_2_ (top) and in the high-symmetry direction of hBN (bottom) (adapted with permission from reference [95]). (d) Scanning Kelvin probe map showing the variation in surface potential over WS_2_ on graphene with a variable number of layers (adapted with permission from reference [101]).

There has been a strong demand for developing direct optical imaging methods that are able to characterize individual defects with nanometer resolution. The simplicity, versatility and non-invasive nature of optical microscopy makes it an indispensable technique in the characterization of 2D materials. To that effect, a sincere focus has been on defect-based single photon quantum emitters in 2D materials. Although point defects are known to localize excitons in TMDs, their direct correlation to quantum emission is not well established [74]. While the early work on hBN has been limited to exfoliated single crystals and identifying conditions to create reproducible defects for single photon emitters, recent work has focused on CVD-grown samples and involved hyper-spectral and nanoscopic imaging using single molecule localization microscopy (SMLM) [75]. It is now possible to resolve these emitters by exploiting the advantages of their blinking properties. The deep-subwavelength (≈10 nm) spatial resolution of this technique allows for resolving two closely located emitters using wide-field imaging at high throughput, while simultaneously being non-destructive or non-perturbative [76] (figure 2(a)). This work highlights the great potential of the SMLM technique for the quantitative, multidimensional mapping of defect properties with applications in material science, quantum information processing and biological imaging. It is worth noting the recent interest in determining the spin properties of defects in hBN for their potential realization as optically addressable spin-qubits [77]. More research activity is likely to be seen on this topic in the coming years. Furthermore, confocal laser scanning microscopy (CLSM) is another example of a non-invasive, rapid, yet easily accessible technique. CLSM provides an optimal balance of image resolution and acquisition time. In a comprehensive comparison study, CLSM showed excellent correlation with conventional optical microscopy, Raman mapping and a variety of scanning probe techniques [78] (figure 2(b)).

### 3.2. Spectroscopic characterization

Raman scattering and photoluminescence (PL) spectroscopy remain two of the most powerful and highly applicable non-contact, non-destructive optical methods for discerning the properties of 2D materials. Raman-based techniques provide information on the chemical, magnetic, electronic and structural properties of 2D materials [79]. An enormous amount of physical information can be extracted and quantified from the Raman spectra, including information about layer thickness, disorder, edge and grain boundaries, doping, strain, symmetry, layer orientation, thermal conductivity, magnetic ordering, and unique excitations such as charge density waves [80]. Raman spectroscopy efficiently probes the evolution of the electronic structure and the electron-phonon, spin-phonon, and magnon-phonon interactions as a function of temperature, laser energy, and polarization. As many TMD 2D materials are particularly vulnerable to optical degradation under ambient conditions, Raman and PL methods are regularly used for studies of degradation in variable environmental conditions and are able to study materials encapsulated in flakes of hBN.

Recently, surface enhanced Raman scattering (SERS) has emerged as a prominent characterization technique. It has been found that TMDs are ideal plasmon-free substrates for SERS due to their high density of states (DOS) near the Fermi level [81, 82]. SERS studies utilizing MoS_2_ and NbS_2_ to probe copper phthalocyanine molecules observed an enhancement factor of up to ≈10^6^ compared to standard Raman performance, where the abundant DOS in NbS_2_ led to the strongest binding energy with the molecules compared with other 2D materials such as graphene or MoS_2_. The large DOS increases the intermolecular charge transfer probability and thus induces significant Raman enhancement [83]. Another prominent direction of Raman spectroscopy is in hyperspectral imaging. This powerful tool can be used to connect spectral and spatial information, where each pixel on the map contains an entire Raman spectrum [84, 85]. The hyperspectral method reveals subtle spatial changes in the characteristic 2D material peaks, which can be used to analyze fine physical effects such as disorder, layer orientation and symmetry. Among other studies, the hyperspectral mapping accompanied by the analysis of the extra peaks in the PL spectra has been successfully applied to study the interlayer radiative recombination of spatially separated carriers in 2D heterostructures in an extended temperature range [86]. Raman spectroscopy has also proved a useful tool for studying strain and doping in 2D materials [87, 88]. Characteristic Raman peaks are affected by both strain and doping, yet the basic Raman analysis does not allow a straightforward separation of the two intertwined effects. Using correlation analysis, however, the effects of strain and charge can be optically separated, enabling their quantification in graphene, MoS_2_ and 2D heterostructures [89–91]. Furthermore, nanoscale variations in strain and doping have been linked to local features and defects in the heterostructures. These features include fractures, folds, bubbles and edges, providing quantitative information and ability to control strain through the local defect structure [92].

Raman-based techniques have been at the forefront of new methods to measure the thermal properties of atomically thin materials and heterostructures, and more recently in hBN. Owing to its wide bandgap, high in-plane thermal conductivity and excellent thermal stability, atomically thin hBN is a strong candidate for heat dissipation applications, especially in the next generation of flexible electronic devices. The nano-second transient thermoreflectance technique has been utilized to verify the tunability of hBN thermal conductivity by controlling the boron isotope concentration. For monoisotopic ^10^B hBN, an in-plane thermal conductivity as high as 585 W m^*−*1^ K^*−*1^ at room temperature has been reported [93]. In addition, optothermal Raman measurements are able to measure the thermal conductivity and thermal expansion coefficients in atomically thin hBN in a suspended geometry. A monolayer of hBN has been found to have a high thermal conductivity of 751 W m^*−*1^ K^*−*1^ at room temperature, which decreases with layer thickness, making it one of the best thermal conductors among semiconductors and electrical insulators [94].

Angle-resolved photoemission spectroscopy (ARPES) and electron energy loss spectroscopy are two key techniques for determining the electronic structure in 2D materials, as well as correlated and topological materials. The modern ARPES tools include complementary spatial/energy/momentum resolutions and *in situ* sample preparation, allowing the relationship between electronic structure and topology to be examined with unprecedented resolution. Recent examples include determining the spatially-resolved electronic structure of WS_2_, graphene, hBN and TiO_2_ heterostructures [95, 96]. Among these findings is a striking renormalization of the spin-orbit splitting of the WS_2_ valence band, which can be controlled by chemical doping or by choice of substrate and is attributed to the impact of trion formation on the self-energy of carriers in WS_2_ (figure 2(c)). Similar modulations and band shifts were reported due to dielectric screening and disorder in the surrounding environment. Another example of an ARPES study is observing modifications in the electronic structure of ‘twisted’ layers of WS_2_ on graphene [97].

### 3.3. Scanning probe techniques

Scanning tunneling microscopy and spectroscopy (STM/STS) are commonly used in fundamental studies of 2D materials, providing a broad spectrum of the highly entangled topographical and local electronic information on the atomic scale. The technique is highly complementary in studies of fine electronic phenomena, *e.g.* local doping, mapping of the bandgaps, local disorder, direct characterization of the band offset at heterostructure interfaces, *etc.* For example, recent studies pointed towards the absence of Fermi-level pinning for vdW 2D TMD/metal heterojunctions, following the prediction of the Schottky–Mott model [98]. Another example is the use of STM for the study of black phosphorus (BP), which is one of the most promising 2D semiconductors due to its layer-dependent bandgap and high mobility carriers. Its puckered crystal structure also creates a unique electronic anisotropy, which creates opportunities for novel angle-dependent electronic and optoelectronic devices. STM allowed for a temperature-dependent study of the native defects (primarily phosphorus vacancies) in BP and led to the observation of a periodic electronic superstructure there [99]. In a recent perspective review article, STM has been highlighted as one of the most important tools for understanding intrinsic and extrinsic disorder associated with atomic defects in 2D materials and heterostructures and thus a highly valuable technique for quality control on the atomic scale, enabling further scientific advances and progress toward technological applications [100].

Other prominent scanning probe techniques include Kelvin probe force microscopy (KPFM), which is widely used for mapping the surface potential in 2D materials as well as identifying the number of layers (figure 2(d)) [101]. For example, KPFM is successfully and commonly used to distinguish between areas of single-, bi- and few-layered graphene. However, standard KPFM does not generally provide reliably comparable values, and significant variations of the parameters have been typically reported [102, 103]. Alternatively, a quantitative approach has been recently demonstrated and allows for reliable direct measurements of surface potential and work function on the nanoscale with traceability to the macroscopic primary standards [102, 104, 105]. KPFM has also been applied to measure the gap states, their energy distribution and Fermi level pinning in MoS_2_ monolayers and multilayer transistors [106]. Furthermore, the electronic properties of 2D materials are highly sensitive to atoms and molecules adsorbed on their surface and to changes in their environment. Being a surface property, the work function can be strongly affected by environmental conditions. KPFM is indispensable to study the effect of environmental conditions (*e.g.* humidity, temperature, specific gas environment) on the work function and doping level of graphene and 2D materials [104].

Scanning nonlinear dielectric microscopy (SNDM) is another interesting technique for imaging of local properties in 2D materials. This microwave-based method has been implemented for imaging the nanoscale carrier distribution in pristine and Nb-doped MoS_2_ [107]. SNDM allowed visualization of nanoscale domains with dominant p- or n-type doping through the detection of differential capacitance induced by a small ac-bias voltage. The results also revealed variations in the nanoscale carrier distribution on the areas with different stacking layers [108].

### 3.4. Probing the properties of 2D magnets

The thickness dependence of the magnetic interactions in CrI_3_ is an intriguing puzzle plaguing researchers since the initial discovery that layers in atomically thin CrI_3_ are AFM stacked, in contrast with the pure FM stacking displayed in the bulk [50, 109]. From recent theoretical and experimental work, it appears that crystal structure is the key. Whereas bulk CrI_3_ goes through a crystallographic phase transition at 220 K from monoclinic to rhombohedral stacking, it has been discovered that atomically thin CrI_3_ instead remains in the monoclinic structure for temperatures down to liquid helium temperatures, where AFM stacking is energetically favorable [110–112].

**Figure 3.**
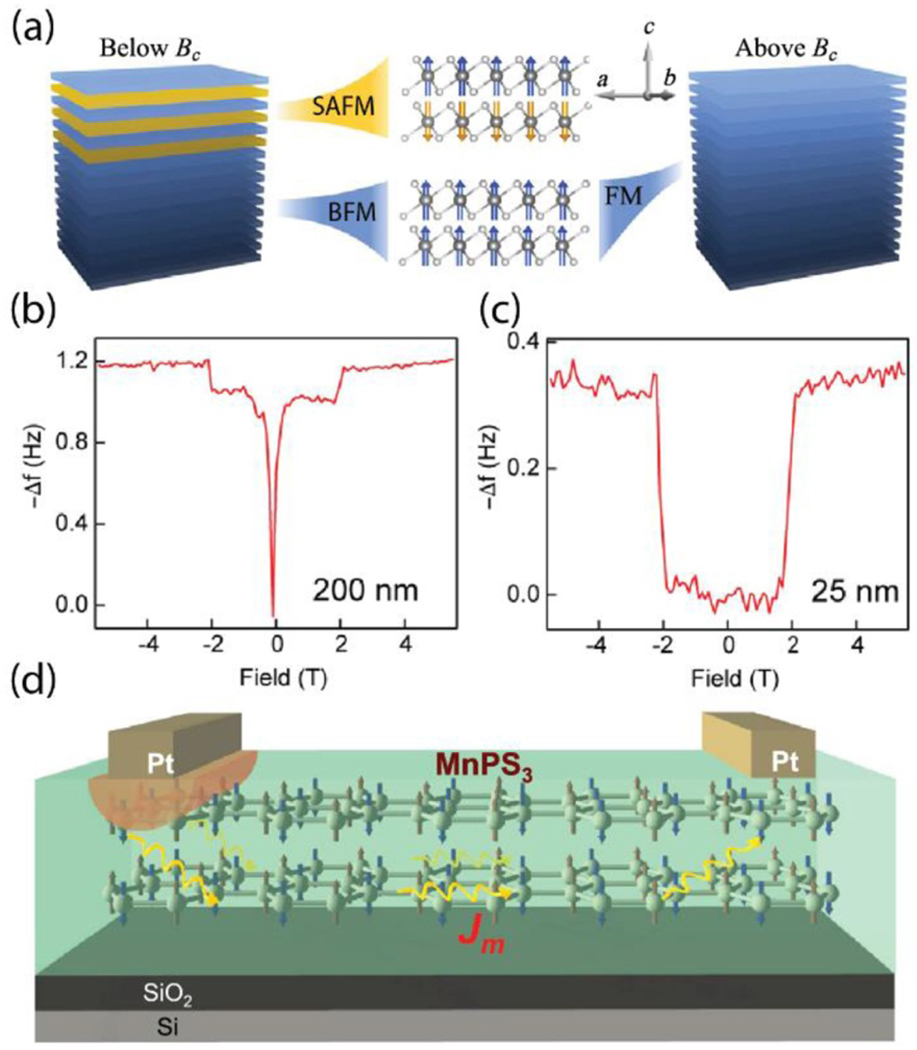
(a) Illustration of surface AFM (SAFM) layers and bulk FM (BFM) layers in bulk CrI_3_ below and above the critical magnetic field B_c_ (adapted with permission from reference [115]). Magnetic force microscopy signal as a function of applied magnetic field along the spin direction for two CrI_3_ flakes with thicknesses of (b) 200 nm and (c) 25 nm. The 200 nm flake shows spin polarization of both the BFM (≈0 T) and SAFM (≈2 T) layers, while samples 25 nm and thinner only show spin polarization of SAFM layers (adapted with permission from reference [114]). (d) A schematic of magnon transport in a quasi-2D antiferromagnetic MnPS_3_ device (adapted with permission from reference [121]).

Interestingly, magneto-tunneling measurements revealed AFM stacking in samples even 20 layers thick, which is significantly thicker than when layer effects no longer dominate in other 2D materials, such as the TMDs [113]. In a detailed study of thickness-dependent (≈100 *μ*m, ≈2 *μ*m, and ≈50 nm) magnetization and critical behavior in bulk CrI_3_, a jump in the magnetization has been observed at B ≈ 2 T in all thicknesses, representing the same flip in the interlayer stacking from AFM to FM that is observed in the atomically thin samples [54, 113]. Since the samples were micrometers thick, and thus assumed to be purely FM, the authors attributed the jumps to a depinning of magnetic domains. Recent magnetic force microscopy and magneto-Raman spectroscopy results, however, have indicated that both FM and AFM stacking orders can co-exist in a bulk flake, with the top ≈13 nm at each surface comprising of AFM coupled, monoclinically stacked layers, while the internal layers are FM coupled with rhombohedral stacking (figure 3(a)) [114, 115]. Thus, samples that are less than 25 nm only display AFM stacking (figures 3(b) and (c)) [114]. This sheds significant light on the origins of the AFM stacking for atomically thin CrI_3_, but further research into what causes the reconstruction of the surface layers and its dependence on crystal growth process and/or exfoliation/encapsulation parameters is needed.

A recent, novel development in the characterization of vdW magnets is the study of magnons, or quanta of spin waves. For 3D bulk structures, one of the most common ways to study magnons is through inelastic neutron scattering. However, the requirement of large sample sizes puts inelastic neutron scattering at a disadvantage, since vdW magnets typically display properties that are strongly correlated to the number of layers, with new physics occurring in the few-layer (few-nm) regime. Thus, researchers have turned to optical techniques, which have diffraction-limited spatial resolution, to probe magnons in the nanoscale regime. Raman spectroscopy as a function of temperature, magnetic field, and polarization has been used to observe and identify one magnon processes in FePS_3_ and CrI_3_ [115–118]. Detailed polarization analysis of the AFM magnon in FePS_3_ challenged previous interpretations that magnons are required to be antisymmetric for these 2D honeycomb magnets [116, 119]. In addition to Raman spectroscopy, ultrafast optical pumping combined with magneto-optical Kerr effect has been employed to probe magnon dynamics in bilayered CrI_3_, demonstrating tunability of magnetic resonance frequency by as much as 40% when varying the gate voltage [120]. Magnon transport on the order of several micrometers has been confirmed for quasi-2D MnPS_3_, suggesting that 2D vdW magnets could provide a material channel for future magnonic devices (figure 3(d)) [121]. While the study of magnons has explored excited spin-states, there has also been novel developments regarding the observation of stable spin-textures in 2D magnets using Lorentz microscopy. Skyrmions and topologically non-trivial spin-textures have been observed in CrGeTe_2_ as well as FeGeTe_2_ and further studies on their manipulation and modulation in device applications are forthcoming [122, 123].

## 4. Novel device applications based on 2D materials

The most promising attributes of 2D semiconductors for many years have been in channel materials for next generation transistors due to their ultra-thin scale that allows superior electrostatic control for deep, lateral scaling. As a result, several attempts have been made at using the semi-transparency of a 2D material to a gate-electric field to enable new functionalities of devices and applications. The discussions lately have been focused on how these materials complement existing device architectures and functions such as Si-based complementary metal oxide semiconductor (CMOS) technology. In addition, challenges in the integration of these materials with such mature technologies have emerged. Recently, significant progress has been made with 2D materials for novel device applications in the following areas:

### 4.1. Logic and memory with 2D materials

**Figure 4.**
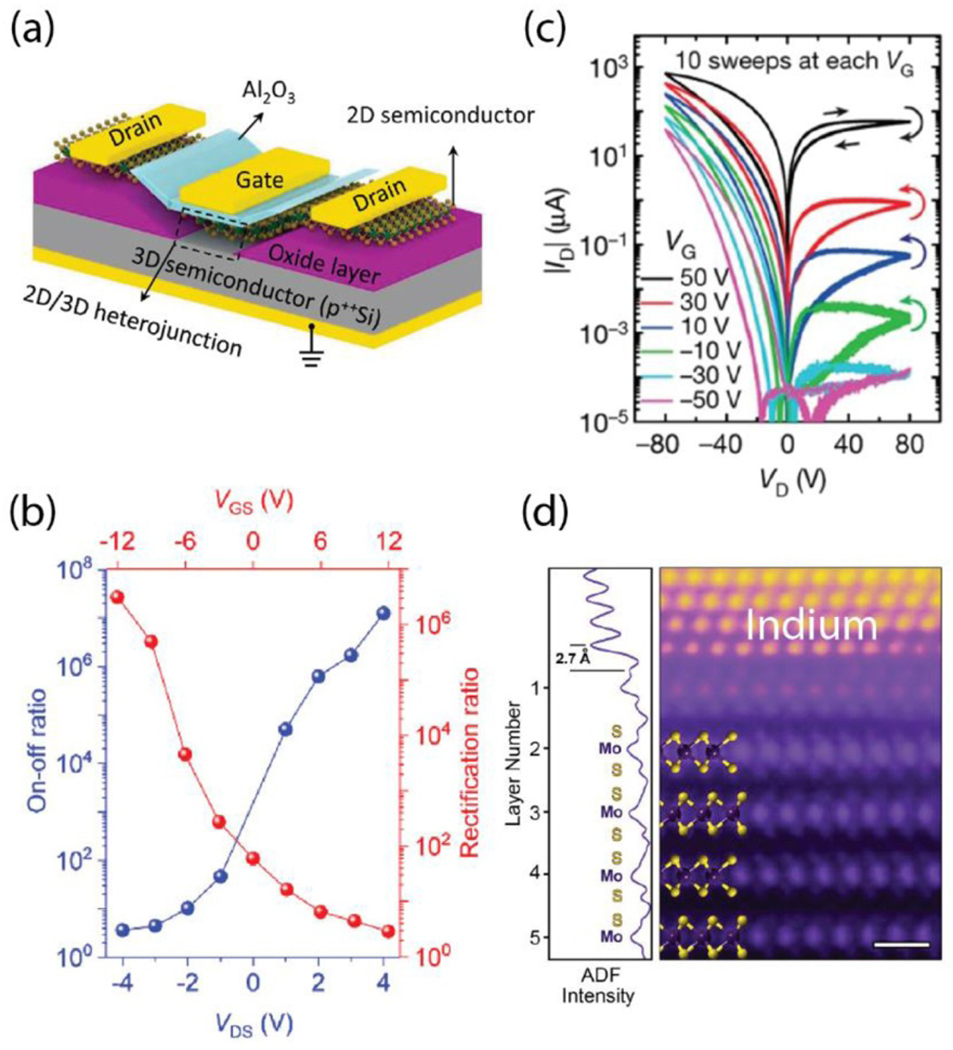
(a) Schematic of 2D/3D gate tunable junctions. (b) ON/OFF ratio (blue) and rectification ratio (red) for MoS_2_/p^++^ Si triode (adapted with permission from reference [124]). (c) Gate-tunable memristive effect in polycrystalline MoS_2_ (adapted with permission from reference [128]). (d) Atomic resolution electron micrograph of In-Au alloy/few layer MoS_2_ interface showing a smooth interface with no basal plane damage (adapted with permission from reference [133]).

Much effort has been involved in the direct integration of 2D semiconductors with conventional Si and III–V semiconductors. This concept presents dual advantages not only in helping to passivate the surface of bulk semiconductors but also by exploiting the complementary and mature substitutional doping schemes of the bulk semiconductors with gate-tunable doping of the 2D integrated materials (figure 4(a)). Such devices have been recently demonstrated in conjunction with solid-state dielectrics showing record performance metrics on sub-threshold swing to ON/OFF current ratios as well as rectification ratios (figure 4(b)) [124, 125]. With a wide range of stable 2D semiconductors now available, exploring and exploiting their combinations with well established, III–V, II–VI and group IV semiconductors will be a fertile playground for logic device innovation for years to come.

While logic devices continue to attract novel ideas, structures and concepts in the context of advancing Moore’s law and Dennard’s scaling rules, a significant amount of recent research in the space of nanoelectronics has been dedicated to non-volatile memory (NVM). This is primarily due to the memory-wall or von Neumann bottleneck faced in modern computing architecture. With the ease of availability of massive amounts of data (Big Data) and the requirement to process it efficiently, the need for power and resource efficient computing is more important than ever. To address the chip architecture and circuit, designers have been researching approaches to in-memory computing, or memory-enhanced computing architectures and denser integration of memory with processors to reduce the von Neumann bottleneck [126]. TMDs have shown significant promise in this regard since 2015 when it was demonstrated that grain boundaries in CVD-grown MoS_2_ exhibited resistive memory effects with changing stoichiometry [127]. This work has been expanded to reveal multi-terminal, gate-tunable, memristive effects that reveal heterosynaptic plasticity as well as several reports on vertical devices (figure 4(c)) [128, 129]. Likewise, electric-double layer gated transistors for NVM memory are also emerging as promising contenders [130]. In particular, approaches using encapsulated molecular monolayers of electrolytes which contain trapped Li^+^ ions have been particularly effective in producing bistable states with long retention times (hours) with ON/OFF ratios of ≈100 [131].

Moreover, the issue for scaling up to large areas with a high degree of uniformity is a persistent challenge. The MOCVD method has become the industry standard for wafer scale growth of 2D materials for electronics. Samsung^5^ has been investigating a variety of 2D materials to complement and enhance the performance of Si technology. They reported that nanocrystalline graphene (ncG) can be grown *via* direct growth on noncatalytic TiN films at temperatures as low as 560 ^*°*^C, which is approaching the back end of line (BEOL) temperature for Si technology. Samsung^5^ has demonstrated application of ncG in interconnects as diffusion barriers to metal silicide formation and as a candidate to interface between metals and Si to reduce the Schottky barrier heights and overall contact resistance in the source and drain region of transistors. Moreover, monolayer MoS_2_ and other 2D materials for applications in triboelectric nanogenerators has been recently revealed as new auxiliary power sources for portable devices. In addition, Interuniversity Microelectronics Center (imec)^5^ is ramping up their 12 inch- (≈305 mm)-wafer platform for beyond graphene 2D materials, assessing the manufacturability of MOSFET devices with MoS_2_ and WS_2_ channels grown by MOCVD. While significant progress is being made on the growth front on non-metallic substrates, the development of reliable transfer techniques from growth to arbitrary substrates is also an essential and emerging development, where imec^5^ is illustrating for the first time an integration flow which is compatible with BEOL requirements. Finally, while the recent progress of materials growth and transfer has certainly been a driving force for research on 2D devices, several problems persist in terms of channel quality, doping and contact resistances. The use of low-boiling point metal contacts such as indium has been recently revealed as a scalable and viable route towards mitigation of basal plane (figure 4(d)) damage due to metallization while concurrently minimizing contact resistance and Fermi-level pinning [132, 133].

### 4.2. Photonics and optics enabled by 2D materials

The optical properties of 2D semiconductors, particularly those of Mo- and W-chalcogenides and hBN, have been equally appealing and represent a highly active area of investigation since the early days. The focus of recent research efforts is exploring the variety of excited states and how they are affected by their surrounding environment, including hybridization with excited states and particles supported in a medium with close proximity, especially in the limit of high-intensity excitation or high exciton density; bi-exciton as well as charged bi-exciton complexes have now been experimentally observed [134, 135]. The charged states have also been subject of electrostatic tunability. From an applied perspective, this electrical tunability of the excitons or their hybridization with another neighboring excitation is equally important. To that effect, recent studies have focused on the use of opto-mechanical and electro-optic coupling to tune excitonic resonances with optical cavity resonances to obtain high ratios of modulation in reflected and emitted light intensities [136]. While excitonic TMDs of Mo and W are attractive, the loss of direct band gap in bilayers and thicker samples is limiting for certain applications, such as light emitting devices. Monochalcogenides of indium, particularly various polytypes of InSe, have recently appeared as optimal 2D semiconductors in this regard [137, 138]. InSe not only possesses a direct band gap in multilayers but can also be tuned by strain and thickness. However, more studies on quantifying and enhancing emission efficiency are desired [139, 140].

Similarly, hybridization of excitons with plasmonic waveguides and photonic-crystal cavity modes in a neighboring Ag single crystal layer separated *via* thin hBN allows electrostatic control of propagating excitations as a proof of principle concept for photonic-information transfer at deep subwavelength scales on a chip [141]. The concept of light trapping is not limited to monolayers of TMDs. In fact, the large optical constants permit multilayers to serve as both the host and cavity medium for excitons, enabling strong light-matter coupling even with low-quality factor modes, resulting in multipartite coupling and >400 meV strong splitting at room temperatures [142]. Aside from experimental observations, the first principles description of excited states in such heterostructured and hybrid systems between excitonic TMDs and metals or organic or other 2D materials has also been improved recently [143].

### 4.3. Photodetectors

**Figure 5.**
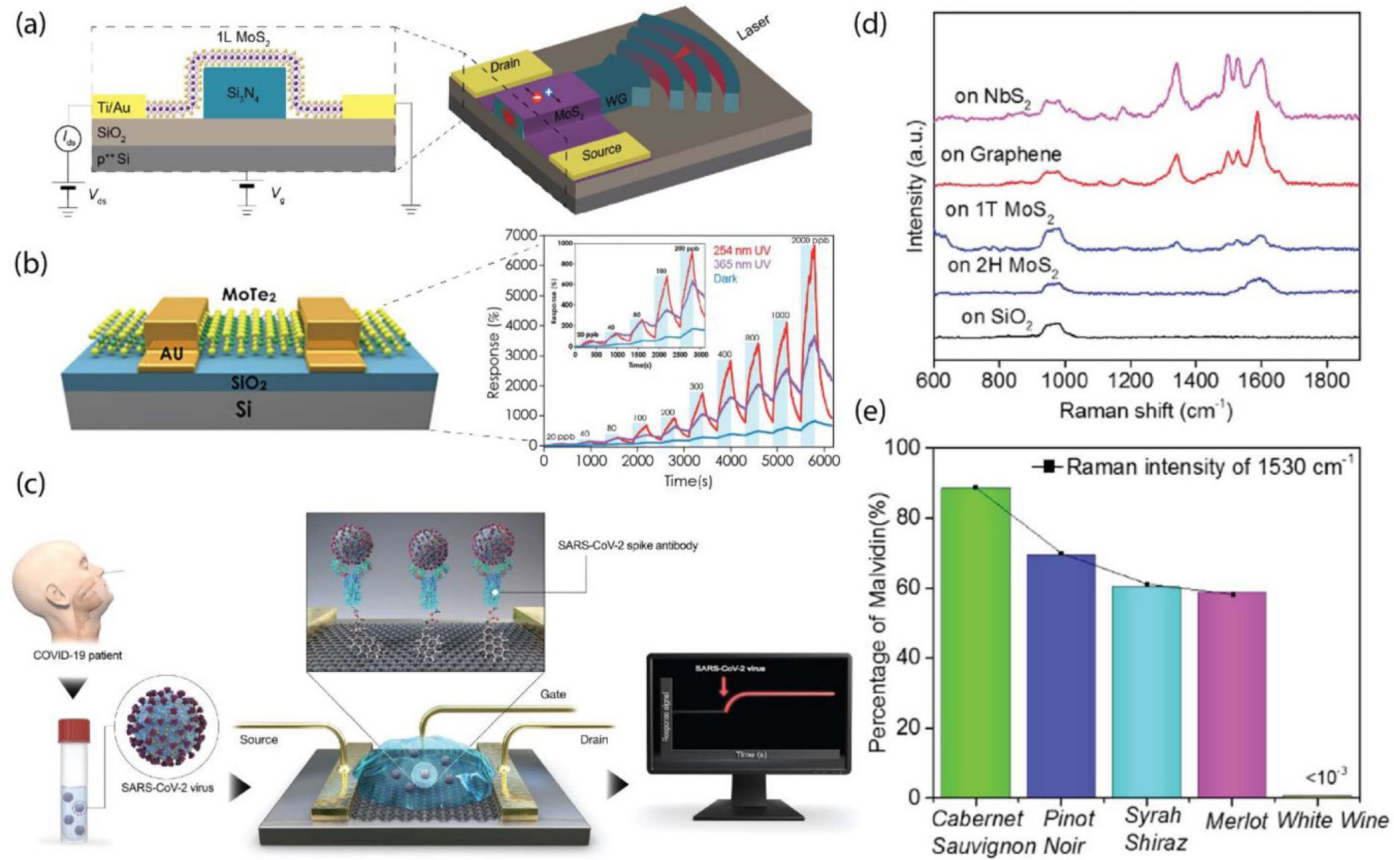
(a) MoS_2_-based photodetector integrated in a photonic circuit. Left: a cross-sectional schematic of the photodetector; right: schematic of light coupling by focusing a laser on the diffraction grating (adapted with permission from reference [144]). (b) Left: Schematic of MoTe_2_ transistor. Right: Dynamic sensing performance toward NO_2_ at concentration from 20 ppb to 2 ppm under different light illumination (adapted with permission from reference [154]). (c) Schematic of COVID-19 FET sensor operation procedure (adapted with permission from reference [175]). (d) SERS effect of 2D materials for red wine detection; top: Raman spectra of Cabernet Sauvignon red wine on blank SiO_2_/Si substrate and different 2D materials; bottom: percentage of malvidin 3-O-glucoside in the analyzed red and white wines based on the Raman intensity using NbS2 as the SERS substrates (black line) and calculated according to the chromatogram results (colored bars) (adapted with permission from reference [83]).

Recent advances in the field demonstrate that 2D crystals with a wealth of exotic dimension-dependent properties can be successfully used in highly sensitive and broad spectral photodetector devices and they are promising candidates for next-generation ultrathin and flexible optoelectronic devices. Specifically, TMDs have emerged as promising materials for photodetection applications due to their strong light-matter interaction, direct bandgap in semiconducting monolayers and compatibility with flexible substrates. Among TMDs, MoS_2_ is becoming prevalent in post Si detectors due to its tunable band gap, which allows a high absorption coefficient and efficient electron–hole pair generation under photoexcitation. Moreover, by using MoS_2_ layers of different thicknesses, photodetection of different wavelengths can be tuned (figure 5(a)) [144]. For example, mono- and bi-layer MoS_2_ were shown to be effective for detecting green light, whereas trilayer MoS_2_ has been well suited for red light detection [145]. Recently, back-gated MoS_2_ p-type FET photodetectors have been demonstrated with UV photodetection capability and rapid response times of 37 ms [146]. The results suggest the potential application of the p-type multilayer MoS_2_ UV photodetectors for environmental and human health monitoring as well as biological analysis. Although photodetectors based on TMDs have shown remarkable performance, devices with high responsivity and detectivity are usually hindered by slow response times. Recently, the photogating mechanism yielding high responsivity in MoS_2_ phototransistors has been explored, where the creation of excitons in the MoS_2_ was linked with desorption of gas adsorbates caused by light illumination [147, 148]. 2D heterostructures have also been intensively studied for applications in photodetectors. A structure combining MoS_2_ films integrated with metallic Mo_2_C multi-gratings provided high responsivity (*R* > 10^3^ A W^*−*1^) and distinctive photodetection (light-to-dark current ratio>10^2^) over a broad spectral range (405–1310 nm) [149]. The grating of metallic Mo_2_C produces plasmonic resonance, delivering hot carriers to the MoS_2_ channel. By adjusting the grating period of Mo_2_C, the optimal photo-response of light can be controlled, from visible to near infrared (NIR).

Beyond TMDs, other 2D materials have been of great interest for the fabrication of photodetectors. BP has shown significant promise for use in infrared photodetectors due to its high carrier mobility, tunable bandgap and anisotropic properties. Although typical BP detectors have been limited to the visible and NIR or single IR wavelengths, recent results demonstrated photoresponses of BP photodetectors across the entire spectral range [150]. These devices show broadband photodetection from ≈400 nm to the nearly 4 *μ*m. In the visible range, a responsivity of >6 A W^*−*1^ has been obtained due to a large photoconductive gain. Beyond BP, a strain-controlled flexible photodetector has been implemented using the piezo-phototronic property of In_1–*x*_Sn_*x*_Se, where the performance of the device can be tuned by applying mechanical strain [151]. The piezoelectric properties and changes in the band gap of the material were systematically studied and the fivefold increase in dark and light drain-source current under a bending strain of 2.7% was demonstrated. This shows a great promise for the design of high performance, multifunctional strain sensors with photodetection capabilities.

### 4.4. Gas sensors

The need to monitor dynamic changes of pollution through gas and humidity sensing has increased the demand for highly sensitive sensor materials. Owing to their unique tunability, 2D materials are excellent candidates for sensing applications. Following the success of graphene, TMDs have become widely utilized for the detection of simple (NH_3_, NO_2_, H_2_, *etc.*) and more complex (*e.g.* various volatile organic compounds (VOCs)) gas molecules owing to their high specific surface area and tunable electronic structures (figure 5(b)) [152–154]. The high surface reactivity of 2D materials offers potential for room temperature gas sensing leading to low power consumption, which is highly desirable for practical applications. However, TMDs are commonly prone to degradation upon exposure to the ambient environment due to significant oxygen adsorption on their surface. This limits the practicability of a sensor in real environment. A solution to this problem is through nanohybrid composites of TMDs. This strategy not only provides the environmental stability, but also improves the overall performance of the sensor.

For example, a highly flexible chemical sensor with enhanced sensitivity that combined multiwall carbon nanotubes (MWCNTs) with WS_2_ and MoS_2_ on cellulose paper with has been reported [155]. The overall approach is simple, scalable, rapid, and cost-effective. Owing to the flexibility of cellulose paper, the sensor enables reversible 3D folding and unfolding, bending, and twisting without any degradation. At the same time, the CNTs form a percolation network and simultaneously provide gas reactivity. Functionalization of CNTs with WS_2_ greatly improves the sensing response to NO_2_ exposure. The sensor maintains high sensitivity even under severe deformation, such as heavy folding and crumpling. Although the reported sensitivity of 4.57% ppm^*−*1^ is much higher than that of previous paper-based NO_2_ sensors, the results have not yet reached the target sensitivities for environmental applications. MoS_2_ is also an excellent material for humidity sensing due to the presence of inherent defects, and high moisture absorption. However, the humidity absorption on the basal plane of MoS_2_ can be limited. Alternatively, 3D nanotubes of MoS_2_ with honeycomb structures synthesized *via* anodic aluminum oxide (AAO)-assisted growth have demonstrated superior humidity sensing accompanied by an ultrafast response [156]. The sensitivity and response speed are enhanced by the open pores of the MoS_2_ nanotubes, leading to higher moisture absorption. MoS_2_ honeycomb structured humidity sensors have shown excellent sensitivity of ≈700% at 83% RH with a fast (sub-second) response and recovery time.

The MXene class of 2D materials has also been applied to gas and humidity sensing, although with a lesser degree than TMDs. TMD/MXene nanohybrids, *i.e.* MoS_2_/Ti_3_C_2_T_x_, have been synthesized using liquid phase exfoliation and inkjet printing, both being cost-effective mass-production methods [157]. The MoS_2_-decorated Ti_3_C_2_T_x_ gas sensor exhibited superior ability to detect extremely low concentrations (1 ppm) of VOCs and more than 10-fold higher response, as compared to a pristine Ti_3_C_2_T_x_ sensor. This results from the synergetic effect of high electrical conductivity of Ti_3_C_2_T_x_ and high specific surface area of MoS_2_ favoring gas adsorption. One can confidently expect more research into this rapidly growing area, which will require reliable testing protocols and the relevant metrological process for sensor traceability and benchmarking.

## 5. Conclusions and future outlook

The interest in 2D and layered materials continues to expand, driven by the compelling properties of individual atomic layers that can be stacked and/or twisted into synthetic heterostructures. Properties that arise from the strong in-plane bonding and weak out-of-plane interactions of individual layers have proven useful for device applications that span electronics, optoelectronics and sensing. These properties include a plethora of electronic band structures with trivial and non-trivial topologies that can be modulated and/or accessed on demand, as well as the emergence of many different quasiparticles, including plasmons, polaritons, trions, excitons and magnons. Furthermore, 2D materials exhibit room-temperature spin and valley polarization, piezoelectricity and strongly coupled properties (magneto- and opto- and electric effects), among others. All of these properties are intricately dependent on the composition, crystal structure, stacking, twist angle, layer number and phases of these materials. There has been a substantial shift in the community on exploring the intriguing properties of 2D magnetism and it is expected to continue to expand. The field has also recently shifted towards studying correlated states of matter and superconductivity in small-angle twisted heterostructures, following the seminal work by Massachusetts Institute of Technology (MIT) on magic-angle twisted bilayer graphene [158, 159], using techniques such as transport, STM, ARPES, *etc.* [160–164]. While most of the work is currently focused on graphene-based twisted structures, new studies involving beyond graphene materials such as TMDs are starting to emerge [165–168].

Initial results on graphene exfoliated from bulk single crystals motivated the development of wide-area, high-purity synthesis and heterojunctions with atomically clean interfaces. Now by opening this design space to new synthetic 2D materials ‘beyond graphene’, particularly with TMDs over the years, it is possible to explore uncharted opportunities in designing novel heterostructures for tunable devices. Moreover, there has been a large interest in 2D ternary alloys, elemental 2D materials and group-III 2D chalcogenides, which have shown many new applications in photonics and electronic devices [169–172]. The already well-established growth schemes of these materials by MOCVD and MBE will surely lead to new discoveries using these materials for years to come. The ability to achieve uniform crystalline heterostructures of any 2D materials combination on a wafer scale is still at an early stage of development. The success of this will be the real turning point to realize and implement these materials in applications that have yet to be explored.

One area that 2D semiconductors will play a key role in is ultralow power devices. As the next era of information technology continues to mature into the era of machine learning and big data analytics, processing this massive volume of data, and at greater speeds, will require at least 100x increase in the density of logic devices. Even more important than logic will be a need for memory devices and tight interation of memory and logic for neuromorphic computing architectures. There is an urgent need for new materials and system level approaches to circumvent the challenges of computation speed. However, at the core of this challenge are also larger issues; energy consumption being the most prominent. The more densely packed integrated circuitry becomes, the more energy is lost in the form of heat due to unavoidable electrical resistance. With no clear path to increase energy efficiency in time to meet the expanding demand for computation speed, the design of the integrated circuit in computers must be revised. 2D materials might hold the solution for the future of computation.

Finally, biosensors fabricated from 2D materials has become a topic of great interest. A wide variety of biosensing schemes, *e.g.* bio-FETs, electrochemical, impedance and fluorescence biosensors, enzymatic biosensing, DNA sensing, and immune sensing have been extensively reported using graphene and other 2D materials [173, 174]. The recent outbreak of the corona virus (COVID-19) has spread globally and posed a major threat to public health worldwide. Reliable laboratory diagnosis has been one of the foremost priorities for promoting public health. As a very quick and efficient response, G. Seo *et al* have demonstrated a graphene-based FET biosensing device for detecting SARS-CoV-2 in clinical samples (figure 5(c)) [175]. The graphene FET was functionalized with a specific antibody against SARS-CoV-2 spike protein. The performance of the sensor was determined using antigen protein, cultured virus, and nasopharyngeal swab specimens from COVID-19 patients. The FET sensor successfully detected SARS-CoV-2 in culture medium and clinical samples, showing excellent limit of detection values, thus demonstrating a highly sensitive and simple immunological diagnostic method for COVID-19 that requires no sample pre-treatment or labelling. Furthermore, graphene oxide provides a remarkable immobilization platform for surface plasmon resonance (SPR) biosensors due to its excellent optical and biochemical properties. The reusable graphene-oxide SPR sensor has three times higher sensitivity than the carboxy-methylated dextran surface of a commercial sensor chip and excellent bioselectivity with more than 25 times reduced binding for nonspecific reactions [176]. This approach was further on expanded using 2D gold nano islands (AuNIs) and implemented for the clinical COVID-19 diagnosis. The method incorporates a dual-functional biosensor combining the plasmonic photothermal effect and localized SPR sensing transduction [177]. The functionalization by complementary DNA receptors allowed for a sensitive detection of the coronavirus 2 (SARS-CoV-2) through nucleic acid hybridization. Such dual-functional localized SPR biosensor exhibited a high sensitivity toward the selected SARS-CoV-2 sequences with a lower detection limit of 0.22 pM and allowed selectivity to the specific target in a multigene mixture. Additionally, 2D metallic materials have been demonstrated a high ability to differentiate various types of red wines by obtaining the direct spectral biomarkers. Such a technology could enable direct, rapid, and reliable detection if commercialized in the future, with applications in agriculture as well as the food and nutrition industries (figure 5(d)) [83].

2D materials offer the ultimate flexibility and scaling potential for device miniaturization, as well as a remarkable platform to study new phenomena in chemistry, materials science, biology and condensed matter physics. We hope that this MRS interdisciplinary symposium continues to bring together a diverse host of researchers to capture the latest developments in synthesis, properties, characterization and applications of ‘beyond graphene’ 2D materials for years to come.

## Footnotes

5 Certain commercial equipment, instruments, or materials are identified in this manuscript in order to specify the experimental procedure adequately. Such identification is not intended to imply recommendation or endorsement by the National Institute of Standards and Technology, nor is it intended to imply that the materials or equipment are necessarily the best available for the purpose.
